# Supplementary material for: Regulation of the Receptor Tyrosine Kinase AXL in Response to Therapy and Its Role in Therapy Resistance in Glioblastoma
Source: Int J Mol Sci. 2022 Jan 17;23(2):982. doi: 10.3390/ijms23020982 (PMC8781963; doi:10.3390/ijms23020982)
Supplement: Supplementary file 1 [file ijms-23-00982-s001.zip › ijms-1532485-supplementary.pdf]

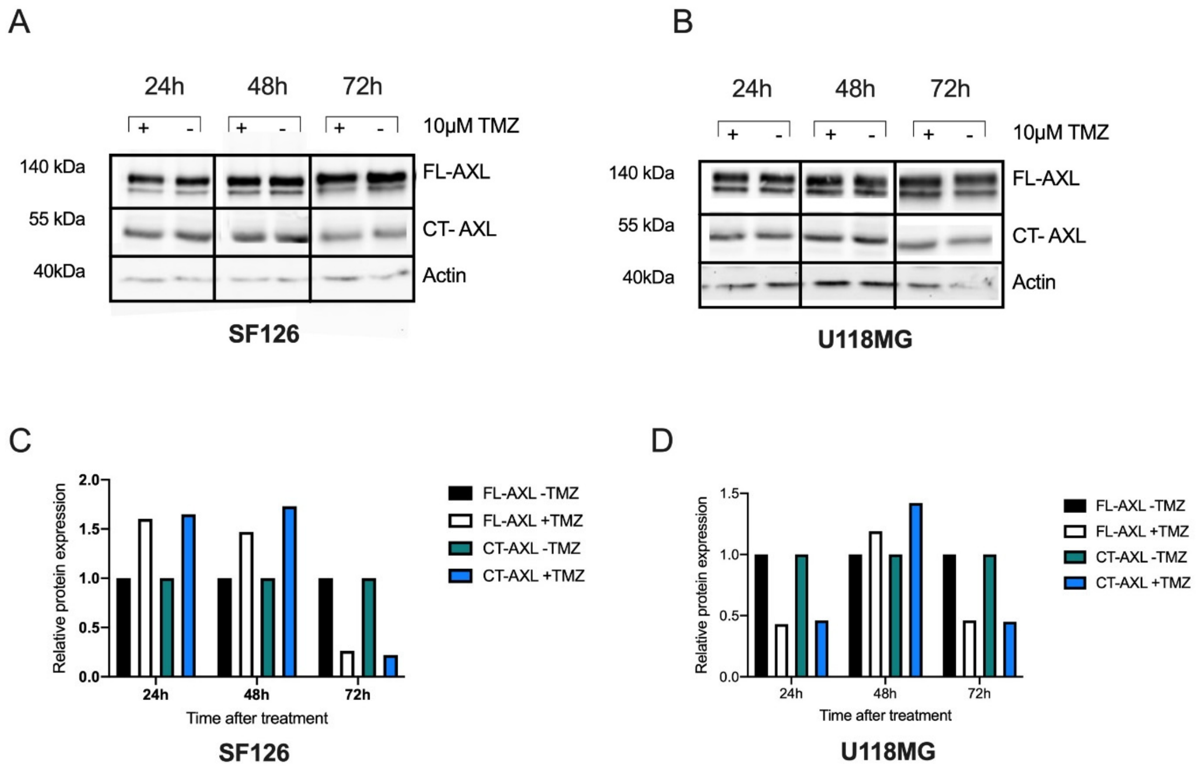

Supplementary Figure S1: A,B: Western blot of FL-AXL and CT-AXL protein for SF126 and U118MG under 24, 48, and 72 h of TMZ treatment. C,D: Quantification of the FL-AXL and CT-AXL protein levels for SF126 and U118MG under TMZ treatment 24, 48, and 72 h (p = ns).

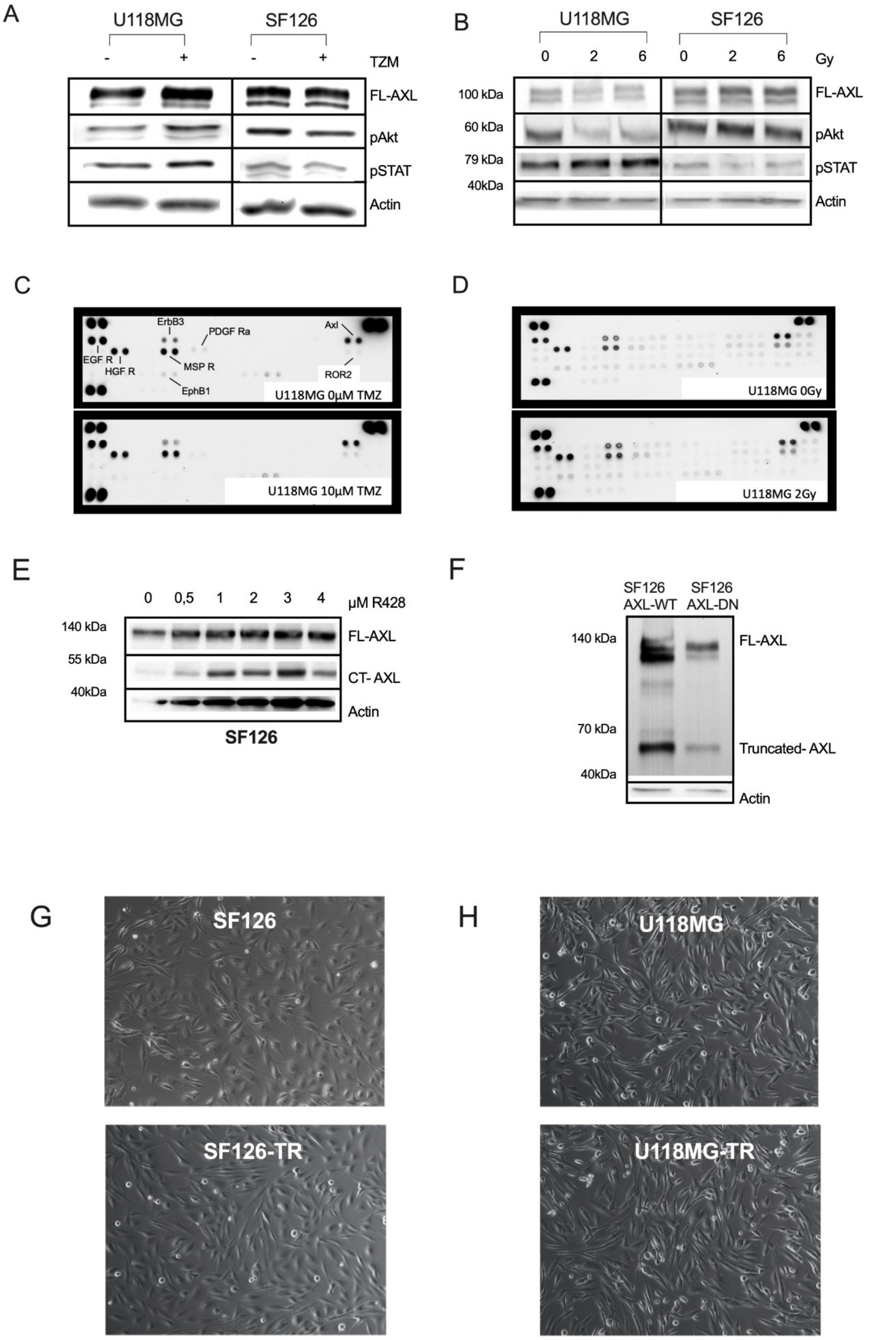

Supplementary Figure S2: RTK-AXL downstream signaling (pAkt, pSTAT) in response to TMZ (A) and RTX (B). C: Proteome Phospho-Kinase Array of U118MG treated without

(upper) and with (lower) 10 µM TMZ. D: Proteome Phospho-Kinase Array of U118 cells treated without (upper) and with (lower) 2 Gy RTX. E: Western blot analysis comparing FL-AXL and CT-AXL expression under TKI R428 in SF126 cell line. F: Western blot analysis comparing the RTK-AXL expression in lysates of SF126 naive, SF126-DN and SF126-WT expressing cell lines and irrespective FL-AXL expression. SF126 glioma cell clones expressing the wild type RTK-AXL receptor (WT) and a truncated dominant-negative mutant form of RTK-AXL ta(AXL-DN). SF126-DN clones demonstrating increased expression levels of the truncated RTK-AXL form at~70 kDa compared with the wildtype. G: 10-fold magni-fication of SF126 and SF126-TR cells and (H) U118MG and U118MG-TR.
